# A299 ACUTE TUMOUR RUPTURE POST-TRANSARTERIAL CHEMOEMBOLIZATION IN A PATIENT WITH HEPATOCELLULAR CARCINOMA

**DOI:** 10.1093/jcag/gwad061.299

**Published:** 2024-02-14

**Authors:** P Parthasarathy, M Khan

**Affiliations:** Gastroenterology, Western University, London, ON, Canada; Gastroenterology, Western University, London, ON, Canada

## Abstract

**Background:**

Hepatocellular carcinoma (HCC) is the most common primary liver malignancy, with increasing worldwide incidence. Transarterial chemoembolization (TACE) is an interventional radiological (IR) therapy used largely in intermediate stage HCC, which can provide successful bridge or downstaging to liver transplantation and increased survival rates. Tumour rupture is a very rare complication of TACE, with an estimated incidence of 0.68%. Potential risk factors include tumour size ampersand:003E 10 cm, and location of lesions in the right lobe or near the liver capsule. However, the pathophysiology of post-TACE tumour rupture is still not fully understood.

**Aims:**

We aim to describe a case of HCC tumour rupture that occurred in the first 30 hours post-TACE. We also aim to highlight the extent of multiorgan ischemia associated with this tumour-related arterial bleed.

**Methods:**

A PubMed literature review was done to ensure that post-TACE tumour rupture was a rare and sparsely reported event. Subsequently, inpatient notes, laboratory and imaging data were obtained from the patient's medical record in an anonymized manner.

**Results:**

A 66-year-old male with alcohol-related liver cirrhosis (MELDNa 7; Child-Pugh Class A) was diagnosed with a 3.3 cm HCC, spanning segments 5 and 8. Six months later, after multidisciplinary discussion, he underwent successful elective TACE of a segment 8 artery that was supplying the HCC, as a bridge to liver transplantation. The next day, he developed diffuse abdominal pain, emesis, tachycardia, and abdominal firmness and tenderness. Labs showed a 45 g/L drop in hemoglobin (121 g/L to 76 g/L), leukocytosis (27.0 x10^9^/L), and an elevated lactate (4.4 mmol/L). Computed tomography (CT) scan confirmed tumour rupture, with moderate-to-large hemoperitoneum with active bleeding from the embolized region of the HCC, and associated gas. Emergent IR-guided embolization was performed to the bleeding segment 8 artery.

CT scan two days later revealed interval ischemic infarction of the spleen, pancreatic tail, and stomach. This widespread ischemia was suspected to be due to hypovolemic hypoperfusion vs. post-TACE embolic phenomena. Aggressive supportive measures, antibiotics, and anticoagulation were implemented.

Due to tumour rupture, the patient was deemed no longer a suitable candidate for liver transplantation. Futility of definitive management was discussed, and the patient opted for medical care without resuscitation. After optimization of nutritional and physical functioning parameters, he was discharged home with medical management for pain and ascites.

**Conclusions:**

Post-TACE tumour rupture is a rare occurrence for which the pathophysiology is not fully understood. This case provides an important reminder of this potential adverse event. Early detection and management are imperative to facilitating a favourable outcome in this life-threatening condition.

**Funding Agencies:**

None

